# Statin Use and the Risk of Developing Graves’ Orbitopathy in Patients With Graves’ Disease: A Systematic Review and Meta-Analysis With Regional and Gender-Stratified Analyses

**DOI:** 10.7759/cureus.94818

**Published:** 2025-10-17

**Authors:** Marjeta Kermaj, Bezalel Hakkeem, Henri Fero, Vijay Kumar Doddapaneni, Agron Ylli

**Affiliations:** 1 Endocrinology, University Hospital Center (UHC) "Mother Teresa" University of Medicine Tirana (UMT), Tirana, ALB; 2 General Practice, Government Medical College Kozhikode, Kozhikode, IND; 3 Critical Care, Department of Medicine, Schulich School of Medicine and Dentistry, Western University, London, CAN; 4 Internal Medicine, St. Vincent Medical Center, Toledo, USA

**Keywords:** asian populations, graves’ disease, graves’ orbitopathy, meta-analysis, statins, systematic review, thyroid eye disease, western countries

## Abstract

Graves’ orbitopathy (GO) is a clinically significant extrathyroidal manifestation of Graves’ disease (GD), often resulting in functional and cosmetic morbidity. Preventive strategies remain limited. Statins, widely prescribed for cardiovascular disease, possess anti-inflammatory and immunomodulatory properties that may also mitigate the risk of GO. This systematic review and meta-analysis aimed to evaluate the association between statin use and GO risk in GD, with subgroup analyses by geographic region and sex to capture potential differential effects. This study was conducted in accordance with Preferred Reporting Items for Systematic Reviews and Meta-Analyses (PRISMA) 2020 guidelines. PubMed, the Excerpta Medica database (Embase), and the Cochrane Library were systematically searched from database inception through May 2025 using a predetermined strategy. Eligible observational studies (cohort or case-control) assessing the effect of statin use on incident GO were included. Where feasible, pooled hazard ratios (HRs) or odds ratios (ORs) were estimated using random-effects models (Western cohorts). For Asian populations, effect estimates were synthesized narratively due to heterogeneity in effect measures. Two reviewers independently performed study selection, data extraction, and risk of bias assessment using the ROBINS-I tool. The protocol was prospectively registered in the International Prospective Register of Systematic Reviews (PROSPERO; registration number: CRD420251032246). Five studies (n = 156,926) met the inclusion criteria. In Asian populations (n = 113,628), statin use was associated with a significantly reduced risk of GO (HR = 0.56; 95% CI: 0.46-0.68; p < 0.001). In Western populations (n = 43,298), pooled analysis suggested a non-significant trend toward risk reduction (HR = 0.76; 95% CI: 0.53-1.10; p = 0.14), with substantial heterogeneity (I² = 60.8%). Among Asian females (n = 83,997), statin use consistently demonstrated protective associations (HRs 0.37-0.66), while male subgroup estimates, derived from a single study, were not statistically significant. Across all studies, risk of bias was rated as moderate, primarily due to confounding and variability in outcome measurement. Statin use appears to reduce the risk of GO among patients with GD, with the strongest protective effect observed in Asian women. Although Western data suggested a protective trend, findings did not reach statistical significance and were limited by heterogeneity. These results highlight the importance of region- and sex-specific investigations. Large-scale prospective cohort studies and randomized controlled trials (RCTs) are warranted to confirm the potential preventive role of statins in GO and to clarify dose-response relationships.

## Introduction and background

Graves’ disease (GD) is the most common cause of hyperthyroidism and is frequently accompanied by Graves’ orbitopathy (GO), an autoimmune inflammatory disorder affecting the tissues around the eyes. Clinically, GO often presents with eye bulging (proptosis), double vision (diplopia), ocular discomfort, and, in severe cases, vision loss. It occurs in approximately 25%-50% of patients with GD and can substantially impair quality of life [1,2]. Despite this burden, preventive strategies for GO remain limited, and current management primarily focuses on immunosuppression after clinical onset [3].

Statins, a widely used class of cholesterol-lowering drugs, are primarily prescribed for dyslipidemia and cardiovascular risk reduction. They also exhibit pleiotropic properties such as anti-inflammatory and immunomodulatory effects [4,5], with recent evidence demonstrating reductions in systemic inflammatory markers and modulation of adhesion molecules [6-8]. These additional mechanisms have sparked growing interest in their application beyond lipid-lowering, particularly in autoimmune conditions. In the context of GD, emerging data indicate that statins may help lower the incidence or mitigate the severity of GO, though findings vary across studies and patient populations [9-13].

The immunopathogenesis of GO is driven by T-cell activation, cytokine secretion, and stimulation of orbital fibroblasts, leading to inflammation and tissue remodeling [14,15]. Statins have demonstrated the capacity to modulate these pathways through their anti-inflammatory and immunomodulatory effects [16,17]. Epidemiologic data further suggest sex-related differences in susceptibility to autoimmune thyroid disease and GO, with females typically at higher risk [11,18], highlighting the importance of sex-stratified analyses in this context. Nevertheless, the degree to which these findings translate into meaningful clinical outcomes, especially across different ethnic groups and between sexes, remains uncertain and warrants further investigation [18-20].

No prior meta-analysis has examined sex- and region-stratified outcomes in this context. This study aimed to systematically review and meta-analyze available cohort studies examining the association between statin use and the risk of developing GO in patients with GD, with pre-specified subgroup analyses by geographic region (Asian vs. Western) and by sex to capture clinically relevant heterogeneity.

## Review

Methodology

**Figure 1:**
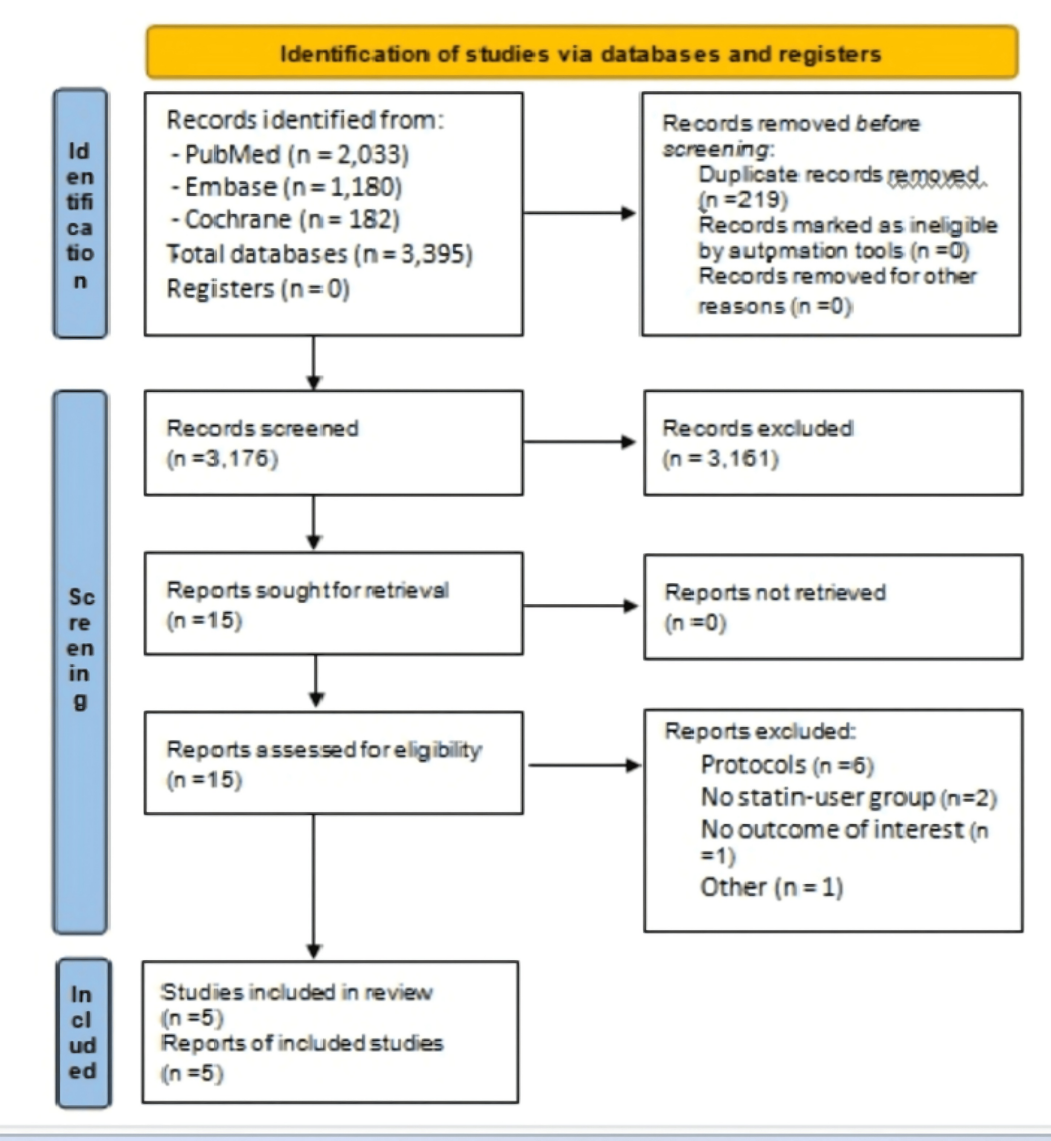
PRISMA 2020 flow diagram of study identification, screening, eligibility, and inclusion Adapted from Page MJ, et al. The PRISMA 2020 statement: an updated guideline for reporting systematic reviews. BMJ. 2021;372:n71. doi:10.1136/bmj.n71. Licensed under CC BY 4.0 [21]. PRISMA: Preferred Reporting Items for Systematic Reviews and Meta-Analyses; Embase: Excerpta Medica database

We conducted this systematic review and meta-analysis in accordance with the Preferred Reporting Items for Systematic Reviews and Meta-Analyses (PRISMA) 2020 guidelines (Figure 1).

Protocol and Registration

The protocol for this systematic review and meta-analysis was prospectively registered with the International Prospective Register of Systematic Reviews (PROSPERO; CRD420251032246). We conducted this systematic review and meta-analysis in accordance with the PRISMA 2020 guidelines [21]. The completed PRISMA 2020 checklist is provided in Appendix A.

Search Strategy

A comprehensive literature search was conducted in PubMed, the Excerpta Medica database (Embase), and the Cochrane Library from database inception to May 2025, utilizing a predetermined search strategy. The search terms included “Graves’ disease,” “Graves’ orbitopathy,” “thyroid eye disease,” “statin,” and “hydroxymethylglutaryl-CoA reductase inhibitors.” Boolean operators and database-specific subject headings (e.g., MeSH, Emtree) were applied. Reference lists of all included studies and relevant reviews were also manually screened to identify additional eligible publications. No language or publication date restrictions were applied. The full electronic search strategy for each database is provided in Appendix B.

Eligibility Criteria

We included observational studies (cohort or case-control) that enrolled patients with GD without pre-existing GO and assessed the association between statin exposure and the subsequent development of GO. Eligible studies were peer-reviewed full-text articles in English from any geographic region. Outcomes had to include incident GO after statin exposure, reported as adjusted or unadjusted hazard ratios (HRs) or odds ratios (ORs), with sufficient raw data for effect estimation.

We excluded non-human studies, reviews, editorials, conference abstracts, duplicate datasets, and studies lacking relevant outcomes. No a priori exclusions were applied based on specific antithyroid regimens or concomitant lipid-modifying agents; these variables were recorded where reported.

Data Extraction

Two reviewers (MK and BH) independently screened the titles, abstracts, and full-text articles using pre-defined eligibility criteria. Any discrepancies were resolved through discussion and consensus. Data were extracted using a standardized form capturing study design, sample size, country, patient demographics, follow-up duration, definition of statin exposure, outcome measures, and adjusted HR or OR with 95% confidence intervals (CIs). 

*Risk of Bias Asse*ssment

Risk of bias in the included non-randomized studies was assessed using the Risk Of Bias In Non-randomized Studies - of Interventions (ROBINS-I) tool, as recommended in the Cochrane Handbook for Systematic Reviews of Interventions (version 5.1.0) [22]. This tool evaluates seven domains of potential bias: confounding, participant selection, intervention classification, deviations from intended interventions, missing data, outcome measurement, and selection of reported results. The ROBINS-I tool was selected as the most appropriate instrument for this meta-analysis, as all included studies were non-randomized cohort designs, in alignment with Cochrane recommendations for bias assessment in observational evidence. Each study was rated as having low, moderate, serious, or critical risk of bias. Two reviewers (MK and HF) independently performed the assessments, resolving any discrepancies through discussion and consensus. All included studies were judged to have an overall moderate risk of bias. Table 1 presents the domain-specific ROBINS-I ratings for each included study.

**Table 1:** ROBINS-I risk of bias assessment for included studies ROBINS-I: Risk Of Bias In Non-randomised Studies - of Interventions; risk categories: low, moderate, serious, critical

Study	Confounding	Selection of Participants	Classification of Interventions	Deviations from Interventions	Missing Data	Measurement of Outcomes	Selection of Reported Results	Overall
Chou et al., 2025 [12]	Low	Low	Low	Moderate	Low	Moderate	Low	Moderate
Lee et al., 2023 [11]	Low	Low	Low	Moderate	Moderate	Moderate	Low	Moderate
Nilsson et al., 2021 [10]	Moderate	Low	Low	Moderate	Low	Moderate	Low	Moderate
Hsu et al., 2024 [13]	Moderate	Moderate	Moderate	Moderate	Low	Low	Low	Moderate
Stein et al., 2015 [9]	Low	Low	Low	Moderate	Low	Moderate	Low	Moderate

Statistical Analysis

A random-effects model with inverse-variance weighting was chosen a priori because clinical and methodological heterogeneity was anticipated across studies. HRs were log-transformed to compute standard errors (SEs) from their 95% CIs, as recommended by the Cochrane Handbook. ORs derived from 2×2 contingency tables were analyzed separately to avoid mixing heterogeneous effect measures (time-to-event vs. binary outcomes).

Because the included studies reported either HR or OR, we performed separate pooled analyses for each metric, avoiding the combination of both in the same meta-analysis. When statistical pooling was not feasible due to limited subgroup comparability, the findings were summarized narratively.

Where necessary, we estimated summary statistics such as means and standard deviations using validated reconstruction methods: Luo et al. [23] for studies reporting medians and interquartile ranges and Wan et al. [24] for those reporting medians and ranges. Statistical heterogeneity was assessed using the I² statistic, with values >50% indicating moderate to high heterogeneity. Leave-one-out sensitivity analyses were conducted to assess robustness. Pre-specified subgroup analyses examined potential effect modification by geographic region (Asian vs. Western) and sex (female vs. male). Forest plots and pooled estimates were generated using Review Manager (RevMan) version 5.4, developed by the Cochrane Collaboration (2020) [25].

Publication bias was not assessed (funnel plots or Egger’s test) because only five studies were included, consistent with Cochrane guidance for meta-analyses with fewer than 10 studies. Quantitative pooling was feasible for Western cohorts reporting HRs, whereas Asian studies were synthesized narratively due to heterogeneity in effect measures and limited availability of adjusted estimates.

Results

A total of five cohort studies met eligibility criteria, representing 156,926 patients with GD. Baseline characteristics of the included studies, covering demographics, statin exposure, and follow-up duration, are summarized in Table 2.

**Table 2:** Baseline characteristics of the included studies EU: Europe; NA: not available; GD: Graves’ disease; St: statin users; Fu: follow-up; GO: Graves’ orbitopathy; USA: United States of America; M: male; F: female * Pooled estimate from median and interquartile range (IQR) using the method of Luo et al. [23]; ** Mean and standard deviation approximated from reported median and range using the method of Wan et al. [24]; † Data extracted from supplementary material (Table S3) of Chou et al. [12]; ‡ Data extracted from supplementary material (Table S5) of Lee et al. [11].

Study	Country (Cohort)	Design	Sample size (N, GD)	Age, mean (SD) years	Female (N/%)	ST users (N/%)	Age in ST, mean (SD) years	Female in ST (N/%)	Follow-up, mean (SD) years
Nilsson et al., 2021 [10]	Sweden (Western cohort)	Nationwide retrospective cohort	34,894	51.1 (18.2)	28,518 (81.8%)	5,574 (16.0%)	64.6 (12.4)	4,171 (74.8%)	4.6 (5.2)*
Chou et al., 2025 [12]	China (Asia)	Nationwide retrospective cohort	102,858	54.2 (10.4)	76,141 (74.1%)	7,073 (6.9%)	62.9 (10.6)	5,254 (74.3%)	5.2 (3.7)*†
Lee et al., 2023 [11]	South Korea (Asian cohort)	Nationwide retrospective cohort	7,192	43.3 (17.8)*	5,049 (70.2%)	739 (10.3%)	NA	521 (70.5%)	8.2 (1.9)**‡
Hsu et al., 2024 [13]	China (Asian cohort)	Hospital-based retrospective case–control	3,578	44.0 (14.9)	2,807 (78.5%)	340 (9.5%)	NA	NA	4.8 (3.3)
Stein et al., 2015 [9]	USA (Western cohort)	Nationwide retrospective cohort	8,404	46.5 (12.6)	6,689 (79.6%)	NA	NA	NA	5.6 (2.3)

Three studies were conducted in Asian populations (Lee et al. [11], Chou et al. [12], Hsu et al. [13]), encompassing 6,927 statin users and 33,083 non-users. Two studies were based on Western populations (Stein et al. [9], Nilsson et al. [10]), with a combined total of 43,298 participants, while sex-specific results from Lee et al. are described in detail in the corresponding subgroup section.

Subgroup Analyses by Geographic Region (Asian vs. Western Populations)

Asian populations: A pooled meta-analysis of the Asian studies was not feasible because of heterogeneity in effect measures (HR vs. OR) and incomplete adjusted estimates. Chou et al. [12] reported a significant protective effect of statins with an adjusted HR (aHR) of 0.66 (95% CI: 0.54-0.82; p = 0.0001). Hsu et al. [13] also found a protective association, reporting an adjusted OR (aOR) of approximately 0.20 in a case-control design. Lee et al. [11] provided only sex-stratified results, showing a marked risk reduction among females (aHR = 0.30; 95% CI: 0.17-0.52; p < 0.001), while the male estimate was non-significant and not numerically reported. Because of these differences and limited reporting, the findings of Asian studies were synthesized narratively rather than pooled (Table 3).

**Table 3:** Summary of Asian studies on statin use and GO HR: hazard ratio; aHR: adjusted hazard ratio; OR: odds ratio; aOR: adjusted odds ratio; GO: Graves’ orbitopathy

Study	Design	Reported Effect	Notes
Chou et al., 2025 [12]	Cohort	aHR 0.66 (95% CI: 0.54 – 0.82); p = 0.0001	aHR; significant protective effect
Hsu et al., 2024 [13]	Case-control	aOR ≈ 0.20	aOR; protective effect
Lee et al., 2023 [11]	Cohort	Female aHR 0.30 (95% CI: 0.17 – 0.52); Male: non-significant (not reported numerically)	Sex-stratified adjusted HRs only; no overall adjusted effect size

**Figure 2:**
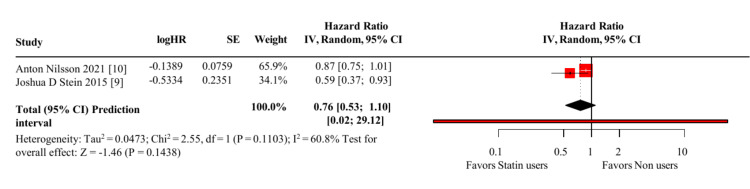
Random-effects meta-analysis of statin use and the risk of Graves’ orbitopathy in Western populations (two cohort studies) The pooled hazard ratio (HR) was 0.76 (95% CI 0.53–1.10); heterogeneity I² = 60.8%; prediction interval 0.02–29.12.

Western populations: A random-effects meta-analysis of the two Western studies (Nilsson et al. [10]; Stein et al. [9]) yielded a pooled HR = 0.76 (95% CI: 0.53-1.10; p = 0.14), indicating a non-significant trend toward a protective effect of statins on GO risk (Figure 2).

Results showed moderate heterogeneity (I² = 60.8%). Nilsson et al. [10] reported HR = 0.87 (95% CI: 0.75-1.01), borderline non-significant, whereas Stein et al. [9] observed a significant reduction (HR = 0.59; 95% CI: 0.37-0.93; p = 0.02). The wide prediction interval (0.02-29.12) highlights the uncertainty of the expected effect in future Western cohorts.

Sex-Specific Subgroup Analysis

Three of the five included studies reported sex-stratified outcomes: two from Asian populations (Lee et al. [11]; Chou et al. [12]) and one from a Western population (Nilsson et al. [10]). In Asian cohorts, both studies found a significant protective effect of statins among females with GD. Lee et al. [11] reported an aHR of 0.30 (95% CI: 0.17-0.52; p < 0.001) in 83,997 women, while Chou et al. [12] observed an aHR of 0.66 (95% CI: 0.54-0.82; p = 0.0001). Neither study demonstrated a significant benefit in males: Lee et al. [11] provided only a visually estimated HR of approximately 0.88 from a supplementary forest plot, not numerically reported and thus excluded from pooling; Chou et al. [12] reported an aHR of 0.88 (95% CI: 0.67-1.14; p = 0.316). Hsu et al. [13] did not present sex-specific estimates. In Western cohorts, Nilsson et al. [10] reported an HR of 0.91 (95% CI: 0.79-1.06) for females, whereas Stein et al. [9] reported only mixed-gender data, preventing subgroup analysis. Due to inconsistent reporting and methodological heterogeneity, a pooled sex-specific meta-analysis was not feasible, and these findings were synthesized narratively (Table 4).

**Table 4:** Sex-specific risk estimates for statin use and GO across included studies HR: hazard ratio; aHR: adjusted hazard ratio; OR: odds ratio; NA: not available; GO: Graves’ orbitopathy Note: * This male HR was visually approximated from supplemental figure S3 from Lee et al. [11] and was not reported numerically; therefore, it was excluded from pooling. All HRs/ORs represent adjusted estimates as reported in the original studies, unless otherwise specified.

Study	Population	Gender-Stratified Outcomes	Female HR/OR (95% CI)	Male HR/OR (95% CI)	Comment
Lee et al., 2023 [11]	Asian	Yes	HR = 0.30 (0.17, 0.52), p<0.001; statin dose (not pooled)	HR ≈ 0.88^*^ (visually estimated, not pooled)	Multivariable Cox regression; HRs reported separately for men and women
Chou et al., 2025 [12]	Asian	Yes	HR =0.66 (0.54-0.82) p=0.0001. Statin users vs non users	HR = 0.88(0.67-1.14),p= 0.3155	Gender-specific aHRs reported
Hsu et al., 2024 [13]	Asian	No	NA	NA	Only overall OR (0.20); no gender breakdown
Nilsson et al., 2021 [10]	Western	Yes (females only)	HR = 0.91 (0.79–1.06)	Not reported	Female-specific HR only
Stein et al., 2015 [9]	Western	No	NA	NA	Mixed-gender outcome only

Discussion

Our meta-analysis shows a protective association between statin use and the development of GO in patients with GD, with the clearest signal in Asian populations, particularly among women. In the female Asian subgroup (n ≈ 83,997), two large studies, Lee et al. [11] and Chou et al. [12], reported consistent and significant risk reduction. In this meta-analysis, we did not pool their crude event counts; instead, we preserved the adjusted sex-stratified HRs from Lee et al. and reported them narratively to avoid mixing adjusted with unadjusted or mismatched effect measures.

These findings align with Barnett et al. [26], who, in a propensity-score-matched cohort of >86,000 patients, reported a 44% reduced hazard of GO among statin users. Although Barnett did not provide sex- or region-stratified data, the direction of effect supports our observed protective signal. Our contribution is the explicit stratification by sex and geographic region, highlighting a stronger protective association in Asian women compared with modest or non-significant effects in Western cohorts.

In Western cohorts, the pooled HR was 0.76 (95% CI 0.53-1.10; p = 0.14; I² = 60.8%), indicating a non-significant protective trend. The inconsistency reflected Stein et al. [9] (HR 0.59, significant) versus Nilsson et al. [10] (HR 0.87, borderline). The wide prediction interval (0.02-29.12) underscores the fragility of the pooled estimate and suggests that future Western cohorts may produce widely divergent effects. Likely drivers include methodological differences (insurance- vs registry-based cohorts), population composition (multi-ethnic U.S. vs largely Swedish), and GO case-definition variability.

Interpretation and Possible Modifiers

Regional and sex-based differences may reflect behavioural and clinical factors (e.g., smoking prevalence, obesity, screening practices), adherence patterns, and statin-prescription differences. Additional biological contributors, including genetic variation, hormonal milieu, and environmental exposures, may influence statin metabolism or immune response [18-20,27-30]. These remain plausible but unproven. Further pharmacokinetic and pharmacodynamic sex differences, together with disparities in prescribing and adherence, may also modulate outcomes [31-33]. For example, sex-related CYP3A4 activity may alter systemic exposure and downstream immunomodulation; however, this hypothesis lacks direct experimental confirmation. We therefore present these as contextual explanations, not definitive mechanisms.

Evidence supporting a preventive role for statins in GO is emerging, including a small randomized trial by Lanzolla et al. [32], a Bayesian network meta-analysis by Xu et al. [33], and a systematic review by Malboosbaf et al. [34]. Our analysis advances this discussion by focusing on the preventive dimension, especially in high-risk subgroups such as Asian women with GD. Socio-economic and racial disparities also influence access to and adherence with statins, while ethnic variation in lipid profiles (e.g., HDL-C, triglycerides) may further modulate response [35-37]. Future studies should routinely collect and report such variables.

Clinical and Research Implications

Given their wide availability and well-established safety, statins could represent a scalable preventive option in GD, particularly for Asian women who already meet cardiovascular indications. However, because current evidence is observational, these associations should be interpreted with caution pending randomized controlled trials (RCTs). Future priorities include RCTs stratified by sex and ethnicity, enrolling newly-diagnosed GD patients with standardized statin exposure and longitudinal GO assessment; large registry-based cohorts; and IPD meta-analyses to resolve subgroup effects, explore dose-response patterns, and integrate genetic, hormonal, environmental, and behavioural covariates.

Study Limitations

This meta-analysis relies entirely on observational data, leaving residual confounding and selection bias. The small study count (n = 5) limits statistical power, especially for subgroup analyses, and precludes formal publication-bias assessment (funnel/Egger). Heterogeneity in exposure definitions, statin type/dose, and reporting prevented a dose-response meta-analysis; only a few studies (e.g., Lee et al. [11]) provided dose-stratified estimates. One retrospective case-control study (Hsu et al. [13]) introduced further methodological heterogeneity. We included only peer-reviewed English-language full texts, which may bias results toward positive findings. These constraints underline the need for protocol registration, inclusion of grey literature, and standardized reporting in future research.

What is Known

GO is a vision-threatening autoimmune complication of GD driven by inflammation and oxidative stress. Statins exhibit anti-inflammatory and immunomodulatory properties and have long been hypothesized as preventive agents. Prior evidence, mostly from retrospective cohorts and one small RCT, suggested a possible benefit; however, previous reviews lacked quantitative synthesis and subgroup analysis.

What This Study Adds

To our knowledge, this is the first meta-analysis to report pooled HRs stratified by both sex and geographic region, revealing a stronger protective association in Asian women and highlighting key methodological gaps (e.g., covariate-adjustment consistency). Our findings emphasize the need for personalized preventive strategies and for rigorously designed, stratified, prospective trials.

## Conclusions

This meta-analysis indicates that statin use may reduce the risk of developing GO, particularly among Asian female patients with GD. The protective association was consistent in Asian populations, whereas findings in Western cohorts were less certain, with moderate heterogeneity and a wide prediction interval. Overall, these results highlight a potential preventive role for statins in high-risk groups, but confirmation from large, sex- and ethnicity-stratified prospective studies or RCTs is needed.

## Appendices

Appendix A 

**Table 5:** PRISMA 2020 checklist Checklist adapted from PRISMA 2020 guidelines [21]. Item numbers correspond to sections of the manuscript. PROSPERO registration: CRD420251032246. PRISMA: Preferred Reporting Items for Systematic Reviews and Meta-Analyses (PRISMA); PROSPERO - International Prospective Register of Systematic Reviews (PROSPERO)

Item	Section/Topic	Checklist Item (shortened)	Location in Manuscript
1	Title	Identify the report as a systematic review	Title page
2	Abstract	Provide a structured summary	Abstract
3	Rationale	Describe the rationale for the review	Introduction (paragraph 1–2)
4	Objectives	Explicit objectives with PICO	Introduction (last paragraph)
5	Eligibility criteria	Study/report criteria	Methods: Eligibility
6	Information sources	Describe all sources + date last searched	Methods: Literature Search
7	Search strategy	Present full search strategy	Methods + Appendix Table 5
8	Selection process	Process for selecting studies	Methods: Study Selection
9	Data collection process	Data extraction methods	Methods: Data Extraction
10	Data items	Variables extracted	Methods: Data Extraction
11	Risk of bias assessment	Methods for risk of bias	Methods: ROBINS-I
12	Effect measures	Principal summary measures	Methods: Statistical Analysis
13	Synthesis methods	Data handling + combining	Methods: Statistical Analysis
14	Reporting bias assessment	Missing results bias	Methods: Statistical Analysis
15	Certainty assessment	Assess confidence	Discussion: Study Limitations
16	Study selection	Numbers screened/excluded	Results: Figure 1
17	Study characteristics	Present study characteristics	Results: Table 2
18	Risk of bias in studies	Present ROB per study	Results: Table 1
19	Results of individual studies	Present study-level results	Results: Tables 1, 3, 4; Fig. 2
20	Results of syntheses	Present meta-analysis results	Results: Subgroup analyses
21	Reporting biases	Assess reporting bias	Discussion
22	Certainty of evidence	Confidence in findings	Discussion
23	Discussion	Interpret results vs evidence	Discussion (paragraph 1)
24	Limitations of evidence	Discuss evidence limitations	Discussion
25	Limitations of review	Limitations of the review process	Discussion
26	Implications	Implications for practice/research	Discussion (final paragraph)
27	Registration & protocol	Registration details (PROSPERO)	Methods: Protocol

Appendix B 

**Table 6:** Detailed database search strategies Full electronic search strategies for PubMed, Embase, and Cochrane Library, as conducted in May 2025. Search terms combined using Boolean operators (AND/OR). Embase: Excerpta Medica database; GD: Graves’ disease

Database	Search Terms
PubMed	("Graves’ disease" OR "Basedow’s disease" OR Graves OR Basedow OR GD OR "Toxic diffuse goiter") AND ("Statins therapy" OR "Hypocholesterolemic agents" OR statin OR atorvastatin OR pitavastatin OR rosuvastatin OR simvastatin OR fluvastatin OR pravastatin OR lovastatin OR "Serum cholesterol" OR "low-density lipoprotein cholesterol") AND ("orbitopathy" OR "ophthalmopathy" OR "thyroid eye disease" OR "thyroid associated ophthalmopathy")
Embase	("graves disease"/exp OR 'graves disease' OR 'basedow disease' OR graves OR basedow OR 'gd'/exp OR gd OR 'toxic diffuse goiter') AND ('statins therapy' OR 'hypocholesterolemic agents' OR 'statin'/exp OR statin OR 'atorvastatin'/exp OR atorvastatin OR 'pitavastatin'/exp OR pitavastatin OR 'rosuvastatin'/exp OR rosuvastatin OR 'simvastatin'/exp OR simvastatin OR 'fluvastatin'/exp OR fluvastatin OR 'pravastatin'/exp OR pravastatin OR 'lovastatin'/exp OR lovastatin OR 'serum cholesterol'/exp OR 'serum cholesterol' OR 'low-density lipoprotein cholesterol'/exp OR 'low-density lipoprotein cholesterol') AND ('orbitopathy' OR 'ophthalmopathy' OR 'thyroid eye disease' OR 'thyroid associated
Cochrane Library	("Graves’ disease" OR "Basedow’s disease" OR Graves OR Basedow OR GD OR "Toxic diffuse goiter") AND ("Statins therapy" OR "Hypocholesterolemic agents" OR statin OR atorvastatin OR pitavastatin OR rosuvastatin OR simvastatin OR fluvastatin OR pravastatin OR lovastatin OR "Serum cholesterol" OR "low-density lipoprotein cholesterol") AND ("orbitopathy" OR "ophthalmopathy" OR "thyroid eye disease" OR "thyroid associated ophthalmopathy")
